# Chloroquine and Sulfadoxine–Pyrimethamine Resistance in Sub-Saharan Africa—A Review

**DOI:** 10.3389/fgene.2021.668574

**Published:** 2021-06-25

**Authors:** Alexandra T. Roux, Leah Maharaj, Olukunle Oyegoke, Oluwasegun P. Akoniyon, Matthew Adekunle Adeleke, Rajendra Maharaj, Moses Okpeku

**Affiliations:** ^1^Discipline of Genetics, School of Life Sciences, University of KwaZulu-Natal, Westville, South Africa; ^2^Office of Malaria Research, South African Medical Research Council, Cape Town, South Africa

**Keywords:** antimalarial drug resistance, chloroquine, malaria, malaria control, sulfadoxine–pyrimethamine

## Abstract

Malaria is a great concern for global health and accounts for a large amount of morbidity and mortality, particularly in Africa, with sub-Saharan Africa carrying the greatest burden of the disease. Malaria control tools such as insecticide-treated bed nets, indoor residual spraying, and antimalarial drugs have been relatively successful in reducing the burden of malaria; however, sub-Saharan African countries encounter great challenges, the greatest being antimalarial drug resistance. Chloroquine (CQ) was the first-line drug in the 20th century until it was replaced by sulfadoxine–pyrimethamine (SP) as a consequence of resistance. The extensive use of these antimalarials intensified the spread of resistance throughout sub-Saharan Africa, thus resulting in a loss of efficacy for the treatment of malaria. SP was replaced by artemisinin-based combination therapy (ACT) after the emergence of resistance toward SP; however, the use of ACTs is now threatened by the emergence of resistant parasites. The decreased selective pressure on CQ and SP allowed for the reintroduction of sensitivity toward those antimalarials in regions of sub-Saharan Africa where they were not the primary drug for treatment. Therefore, the emergence and spread of antimalarial drug resistance should be tracked to prevent further spread of the resistant parasites, and the re-emergence of sensitivity should be monitored to detect the possible reappearance of sensitivity in sub-Saharan Africa.

## Introduction

Malaria is a global health concern regarding morbidity and mortality, with approximately 228 million worldwide cases and an estimated 405,000 deaths in 2018 (World Health Organisation (WHO), 2019). Underprivileged, rural populations consisting of young children and pregnant women are disproportionately affected by malaria. The cornerstone of malaria control efforts for the past decade has been to address the inequalities, including the availability of commodities (Taylor et al., 2017). Sub-Saharan Africa experiences the greatest burden of the malaria disease, accounting for approximately 90% of the world’s *Plasmodium falciparum* infections and deaths with almost all malaria caused by *P. falciparum* in this area (Yeka et al., 2012; Maitland, 2016). *P. falciparum* is the most virulent of the malaria parasites that infect humans (Recker et al., 2018). Therefore, the utilization of effective tools for malaria control has increased to lessen the burden of malaria (Greenwood et al., 2005). In spite of increased efforts, many countries in Africa are still confronted with challenges with malaria control, partially as a consequence of the identified limitations in public health structures as well as the infrastructure of primary health care (Xia et al., 2014). The development of resistance toward antimalarial drugs is one of the main challenges when dealing with malaria control and elimination, as such antimalarial drug resistance in a setting where access to health care is limited has severe consequences (Takala-Harrison and Laufer, 2015). Drug pressure, which refers to the extensive and/or misuse of antimalarial drugs, is an identified factor associated with the emergence of resistance; additionally, the misuse or sole use of a drug can encourage selection of resistant strains (Hanboonkunupakarn and White, 2016).

In Africa, malaria control programs rest majorly on vector control and the use of antimalarial drugs (Conrad and Rosenthal, 2019). There are different types of malaria, namely, asymptomatic, uncomplicated, and severe malaria. Asymptomatic malaria refers to malaria parasites being present in the blood, providing a reservoir for transmission, without the individual displaying symptoms (Chourasia et al., 2017). Uncomplicated malaria refers to malaria symptoms presented by a patient together with a positive parasitological test (Grobusch and Kremsner, 2005). Severe malaria is defined by positive parasitological test (microscopy or rapid diagnostic test) detecting *P. falciparum* and at least one condition for severe disease such as severe anemia or respiratory distress (Conroy et al., 2019).

Sub-Saharan African countries used chloroquine (CQ) as the first-line drug for malaria up to the start of this millennium. However, it was replaced with sulfadoxine–pyrimethamine (SP) after CQ was declared ineffective as a result of resistant parasites (Lusingu and Von Seidlein, 2008) but soon lost its efficacy when parasites began to develop resistance toward it. Mutations in the *P. falciparum* CQ-resistant transporter (*pfcrt*) gene and the *P. falciparum* multidrug-resistance gene 1 (*pfmdr-1*) have been implicated in CQ resistance (CQR) (Bin Dajem and Al-Qahtani, 2010). CQR was observed on the Thai-Cambodian border and concurrently in South America in the late 1950s (Young and Moore, 1961; Harinasuta et al., 1965). In Southeast Asia, specifically Thailand and Myanmar, drug resistance in local parasite populations is a serious concern, with *P. falciparum* in the region tending to develop resistance (Parker et al., 2015). Key factors associated with drug resistance in these regions is drug pressure, which results in selection of resistant parasites and their propagation by local transmission and reservoir migration (Wernsdorfer, 1994). In 1978, resistance spread to Africa, with confirmed treatment failures in both Kenya and Tanzania, later reaching West Africa in 1980. CQ continued to be the first-line treatment for uncomplicated *P. falciparum* malaria in the most sub-Saharan countries until after 2,000 despite its declining use (Flegg et al., 2013). However, some countries, including South Africa, Zambia, Tanzania, and Kenya (D’Alessandro and Buttiëns, 2001; Mwendera et al., 2016), were only forced to switch to SP as the new first-line treatment for malaria, as levels of CQR increased (Gatton et al., 2004). Shortly after its introduction as the new first-line treatment for malaria, polymorphisms in the parasite’s genes—*pfdhps* [*P. falciparum* dihydropteroate synthase (DHFR)] and *pfdhfr* [*P. falciparum* dihydrofolate reductase (DHPS)]—became widespread throughout Africa (Winstanley et al., 2004). The association of the mutations with SP treatment failure in children made the antimalarial unsuitable for therapy (Desai et al., 2015). In Africa, the use of SP for clinical malaria was stopped as a consequence of its extensive resistance. However, in malaria endemic areas, it is still used for intermittent preventative treatment during pregnancy (Zhao et al., 2020). By 2007, 90% of sub-Saharan Africa had implemented policies of artemisinin-based combination therapy (ACT) for the treatment of uncomplicated malaria as a consequence of widespread CQ and SP resistance (Frosch et al., 2011). The efficacy of ACTs leads to substantial declines in morbidity and mortality in areas with high malaria endemicity. But the success of such therapies is threatened by the appearance of artemisinin-resistant strains of *P. falciparum* from Thai-Cambodian and Thai-Myanmar borders (Ajayi and Ukwaja, 2013). There have been isolated reports of artemisinin-resistant parasites in sub-Saharan Africa, but they have not become established on the continent yet (Lu et al., 2017a). The emergence and spread of artemisinin-resistant parasites would have an immense impact on Africa’s control efforts (Raman et al., 2019), especially as the world is anticipating a global elimination strategy (World Health Organisation (WHO), 2020). *P. falciparum* contains Kelch13 (*K13*), which is needed in the asexual erythrocytic development stage. Although details of its function are not fully known (Siddiqui et al., 2020), they are highly conserved, and single-point mutations are associated with artemisinin resistance (Birnbaum et al., 2020). According to Meshnick (1998), the mechanism of action of artemisinin is in two steps: first, an initial activation that catalyzes the cleavage of endoperoxide produces free radicals. In the second step, these free radicals are responsible for killing the parasites. However, a single-nucleotide polymorphism (SNP) in the gene associated with up-regulated pathway antagonizes the artemisinin oxidation activity (Fairhurst and Dondorp, 2016). A recent study revealed that parasites with inactivated *K13* or its mutated form displayed reduced hemoglobin endocytosis (Birnbaum et al., 2020). *K13* is important in the uptake and degradation of hemoglobin, which is vital for parasite survival. The mutations and mislocalization of *K13* induce artemisinin resistance (Xie et al., 2020); therefore, a close observation of ACT resistance is necessary, as they are the current first-line treatment for *P. falciparum* malaria (Siddiqui et al., 2020).

A key challenge for malaria control is the emergence and spread of antimalarial drug resistance, particularly in the malaria endemic areas of sub-Saharan Africa where disastrous consequences are observed as a result of the spread of CQ- and SP-resistant *P. falciparum* strains (von Seidlein and Dondorp, 2015). Furthermore, the development of ACT resistance threatens the control efforts in malaria elimination. This review aims to track the spread of CQ and SP resistance in sub-Saharan Africa. It identifies malaria control strategies employed and ways to combat the challenges being faced to further prevent the emergence and spread of resistant parasites.

## Malaria Control in Sub-Saharan Africa

A global public health concern of this century includes controlling vector-borne diseases such as malaria; therefore, much effort has focused on the development of vector control approaches (Tizifa et al., 2018). These approaches encompass methods directed toward the malaria vector by restricting its ability to transmit malaria by shielding areas that are recognized as receptive areas for transmission. Receptivity of the disease is dependent on the vectorial capacity of local vector populations, besides the presence of the vector; this includes the vector population size, biting habits, and the longevity of the sporogony period. These parameters are greatly affected by local ecology, climate, and human and vector behaviors (Smith Gueye et al., 2016). Examples of vector control strategies include insecticide-treated nets (ITNs) and indoor residual spraying (IRS).

In Africa, ITNs are the most extensively used intervention for malaria control, signifying the main tool for vector control in almost all malaria endemic African countries (World Health Organisation (WHO), 2017). ITNs act as a direct barrier to mosquito biting, hence proving effective in the reduction of malaria-related morbidity and mortality. Additionally, they reduce vector density and the average life span by providing community-wide protection through the killing of mosquitoes (Scates et al., 2020). They exploit the indoor feeding and resting behavioral patterns displayed by some *Anopheles* mosquitoes (Steinhardt et al., 2017). The universal coverage of ITN distribution, with mass distribution campaigns conducted in intervals, is implemented by most national malaria control programs (World Health Organisation (WHO), 2017). More than 800 million ITNs have been distributed in sub-Saharan Africa between 2011 and 2016, to ultimately achieve universal coverage (Olapeju et al., 2018). Through this initiative, a greater proportion (30% in 2010 to 54% in 2016) of Africans in malaria endemic areas slept under ITNs (Olapeju et al., 2018). In sub-Saharan Africa, 67–73% of the total 663 million prevented malaria cases in the past 15 years have been credited to the widespread distribution and use of ITNs (World Health Organisation (WHO), 2017). Another main method used for malaria control on a large scale includes the spraying of houses with insecticides, referred to as IRS. This method has aided the elimination of malaria from large parts of Latin America, Europe, Russia, and Asia. There are also successful IRS programs implemented in parts of Africa (Pluess et al., 2010). It is believed to function by repelling mosquitoes from entering houses as well as through killing female mosquitoes that rest inside houses after taking up a blood meal, thus suggesting that IRS is largely effective against endophilic mosquitoes (those species resting indoors). Additionally, this method is reliant on the vectorial mass effect, which refers to the reduction in transmission as a result of increased mortality of adult vectors typically after feeding (Najera and Zaim, 2001). Considering the slow development and implementation of alternative interventions, ITNs and IRS continue to be the foundation of the malaria control agenda. Consequently, a great challenge is the optimization of the continued use and sustained success of existing ITNs and IRS, while new vector control tools are being studied (Okumu and Moore, 2011).

Antimalarial drugs are used as a malaria control strategy to essentially reduce transmission. During the 20^th^ century, CQ, a safe and inexpensive antimalarial, was a pillar of malaria control and eradication; however, it has lost its effectiveness. Of late, SP was the only alternative extensively available, but the increase and spread of drug resistance have compromised its effectiveness. Resistance to CQ and SP appeared in Southeast Asia, thereafter, spread to Africa (Naidoo and Roper, 2010). Therefore, the World Health Organization (WHO) recommended the implementation of policies approving ACTs as the primary treatment strategy for uncomplicated malaria in the majority of malaria endemic countries (Frosch et al., 2011). In spite of this, according to the 2008 World Malaria Report, ACTs were used to treat only 3% of children with suspected malaria (World Health Organisation (WHO), 2008), implying that CQ and SP were still used to treat malaria in children. Although ACTs are the recommended first-line treatment for malaria in both Asia and Africa, artemisinin-resistant *P. falciparum* strains have appeared and spread within Southeast Asia, subsequently leading to a reduction in treatment efficacy (Dondorp and Ringwald, 2013). Since Africa holds the greatest burden of malaria, there is a concern regarding the impact of artemisinin resistance on malaria morbidity and mortality (Slater et al., 2016).

## The Mechanisms and Contributing Factors Associated With Antimalarial Drug Resistance in *Plasmodium falciparum*

Drug resistance is a growing problem in the fight to control malaria, as pathogens regularly develop mechanisms that allow them to survive the use of drugs. These mechanisms are generally the result of mutations that affect the drug’s target site, thereby hindering or completely preventing binding between the drug and its target (Table 1). Another way that drug resistance may occur is by the increased levels of the target—this means that more drug is needed to reach inhibition of the parasite.

**TABLE 1:** Summary of chloroquine- and sulfadoxine–pyrimethamine-resistant genes, their mutation sites, site, and mode of action.

**Antimalarial and the stage it targets**	**Resistance gene(s)**	**Mutation site**	**Site of action**	**Mode of action**
**Chloroquine Stage of lifecycle—erythrocytic asexual stages (**Lehane et al., 2012)	*Pfcrt* [located on chromosome 7 (Ikegbunam et al., 2019)]	Polymorphism at position 76 (K76T) in the first transmembrane domain (Pulcini et al., 2015)	Digestive vacuole (Shafik et al., 2020)	Mutant *Pfcrt*-mediated CQ efflux lessens access of CQ to its heme target (Ecker et al., 2012)
	*pfmdr-1* [located on chromosome 5 (Ikegbunam et al., 2019)]	The amino-terminal mutations N86Y and F184Y—common to Asian and African parasites (Veiga et al., 2016). The 3 carboxy-terminal mutations S1034C, N1042D, and D1246Y—common to South American isolates (Veiga et al., 2016).	Digestive vacuole (Reiling et al., 2018)	Acts as an auxiliary mechanism alongside diffusion for drug entry into the digestive vacuole (Ibraheem et al., 2014)
Sulfadoxine–pyrimethamine **Stage of life cycle—liver and blood stages (**Raphemot et al., 2016)	*Pfdhfr* [located on chromosome 4 (Fortes et al., 2011)]	Amino acid point mutations at codons N51I, C59R, S108N, and I164L (Jiang et al., 2019)	Folate metabolic pathway (Sharma et al., 2015)	*Pfdhfr*—associated with pyrimethamine resistance (Sharma et al., 2015) *Pfdhps*—associated with sulfadoxine resistance (Sharma et al., 2015). These mutations reduce the binding affinity of SP to the targeted enzymes (Kümpornsin et al., 2014)
	*Pfdhps* [located on chromosome 8 (Fortes et al., 2011)]	Amino acid point mutations at codons S436A, A437G, K540E, A581G, and A613S (Jiang et al., 2019)	Folate metabolic pathway (Sharma et al., 2015)	

*CQ, chloroquine; SP, sulfadoxine–pyrimethamine.*

Chloroquine functions by accumulating in the parasite’s acidic food vacuole (Lehane et al., 2012; Lawrenson et al., 2018). The xenobiotic inhibits heme catabolism (i.e., the mechanism by which the malaria parasite “feeds”). Upon infection, host hemoglobin is degraded by the parasite; this releases heme and leads to the ultimate development of an acidic lysosome-like digestive vacuole (Coban, 2020). Downstream, there is accumulation of heme–CQ complexes, which negatively impact parasite survival (Loria et al., 1999; Lehane et al., 2012).

CQ is able to move through biological membranes and accumulate in the acidic digestive vacuole (Ehlgen et al., 2012). This digestive vacuole functions to conduct proteolysis of hemoglobin, which produces dipeptides and ferriprotoporphyrin IX (a substance that is toxic to the parasite at high concentrations). The parasite biocrystallizes ferriprotoporphyrin IX to hemozoin—this is inhibited by use of CQ (Reiling et al., 2018). CQR arises when CQ cannot accumulate at this active site to break the parasite’s hemoglobin degradation cycle that *Plasmodium* needs (Loria et al., 1999).

CQR is often associated with genes *pfcrt* and/or *pfmdr-1*. The *P. pfcrt* gene is 424-amino acid long and is made up of 10 predicted transmembrane domains (Griffin et al., 2012). It is expressed at all infected erythrocyte stages with maximal expression at the trophozoite stage (Roepe, 2011). In its natural state, CQ is able to permeate the membrane of the food vacuole but becomes protonated in the vacuole and cannot pass the membrane to exit the vacuole (Homewood et al., 1972; Chinappi et al., 2010). As a result, there is a collection of CQ in the vacuole that binds to heme.

However, mutation on the gene increases export of CQ molecules. It has been reported that mutations in *pfcrt* lead to CQ and hydrogen ions being transported out of the food vacuole, which is where CQ exerts its effects from (Lehane and Kirk, 2008; Ecker et al., 2012). As a result, CQ is not activated and does not perform as it should. This is seen phenotypically as CQR.

Gene *pfmdr-1* has also been implicated in CQR. It mediates the production of P-glycoprotein homolog 1. The protein is localized to the food vacuole membrane (Cowman et al., 1991; Njokah et al., 2016). It is a member of a family of proteins that couple ATP hydrolysis to translocation of solutes across cell membranes (Cowman et al., 1991). Structural modeling of *pfmdr-1* assesses biophysical mechanisms of this gene and its protein in conferring resistance with respect to aminoquinoline (Ferreira et al., 2011). It has been established that *pfmdr-1* is involved in transporting xenobiotics (such as CQ) to the food vacuole. To do this, it is located along the food vacuole membrane and pushes CQ away off the cytosol (Dorsey et al., 2001; Ibraheem et al., 2014). The binding domain is on the cytosol-facing side of the food vacuole, allowing it to first encounter antimalarials—this side is where most resistance-causing mutations occur (Ibraheem et al., 2014). Mutations in *pfmdr-1* prevent movement of antimalarials from cytosol into the food vacuole—this reduces potency of some drugs such as CQ, which act in the food vacuole, but drugs that inhibit targets outside the food vacuole can become more potent (Wicht et al., 2020).

SP is an antifolate that is routinely used to treat uncomplicated malaria (Terlouw et al., 2003). Sulfadoxine (SDX) inhibits the activity of DHFR, and pyrimethamine (PYR) inhibits DHPS; these constituents are active against asexual erythrocytic stages of *P. falciparum* (Sandefur et al., 2007). Both DHFR and DHPS are central in parasite metabolism. Specifically, these enzymes are in the folate metabolic pathway. At the end of the pathway, reduced folate cofactors are produced, which are needed for DNA synthesis and metabolism of particular amino acids (Hyde, 2009).

Dihydrofolate reductase is the third enzyme involved in the folate-synthesis pathway in which it combines pteridine with *para*-amino benzoic acid to form dihydropteroate (Heinberg and Kirkman, 2015). SDX is a structural analog of *para*-amino benzoic acid, allowing it to inhibit the DHPS enzyme through competitive inhibition (Nzila, 2006). PYR is a competitive inhibitor of DHFR (Sandefur et al., 2007); thus, in its presence, the folate-metabolism pathway is halted or made less effective. Both SDX and PYR function through competitive inhibition of different targets. In combination, two different enzymes of the same pathway are disrupted and present as resistance to SP.

## The Spread of Chloroquine Resistance in Sub-Saharan Africa

**FIGURE 1:**
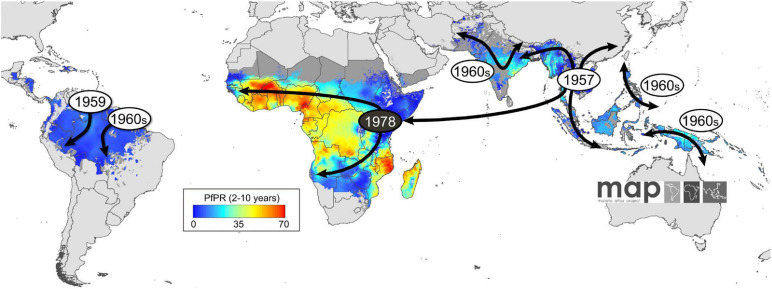
The appearance and global spread of chloroquine resistance (CQR) in *P. falciparum*. Resistance is thought to have arisen in at least six independent origin (gray circles) and moved progressively as a CQ-driven selective sweep, including from Asia to Africa, where it established itself on the East coast in the late 1970s (black circle). The geographic spread of CQR is overlaid onto a current map of *P. falciparum* endemicity modeled for 2010. This map was derived from *P. falciparum* parasite rate (PfPR) surveys, age standardized to the 2- to 10-year age range, using model-based geostatistics (Ecker et al., 2012).

Introduced in the mid-1940s, CQ became the most extensively utilized therapeutic antimalarial drug by 1950. Quinine was essentially swapped out for CQ in areas such as tropical Africa, which lacked systematic malaria eradication programs, therefore making it a pillar of presumptive and mass treatment in eradication campaigns (Nuwaha, 2001). CQ was used as the main malaria treatment therapy up to 1990, owing to its safety, efficacy, and low cost. However, CQR emerged in various parts of the world and quickly spread to West Africa in the 1980s and 1990s (Dagnogo et al., 2018) (Figure 1). Treatment failure, particularly in children who are too young to have acquired immunity, in malaria endemic regions is attributed to the presence of CQR (Trape et al., 1998). CQR is primarily related to an SNP in *pfcrt*, thus leading to an amino acid mutation from threonine to lysine at codon 76 (wild-type K to the mutant T-K76T) (Fidock et al., 2000); additionally, SNPs in exons 2, 3, 4, 6, 9, 10, and 11 of the *pfcrt* gene display possible association with CQR (Awasthi and Das, 2013). Three main haplotypes occur in codons 72–76 of *pfcrt*, thus resulting in the wild-type CVMNK and CQR haplotypes CVIET and SVMNT. In Africa, the most dominant mutant haplotype is CVIET (Thomsen et al., 2013). Another mutation conferring resistance to CQ is the N86Y allele of *pfmdr-1* (Ebel et al., 2020). The role of *pfmdr-1* mutations (N86Y, Y184F, S1034C, and D1246Y) in facilitating *in vitro* and *in vivo* CQR has received a lot of interest in research (Oladipo et al., 2015). CQR advances in three particular ways in each newly affected country, including its increasing spread over a number of locations and regions, the increased prevalence of resistant strains in each area, and the increase in the intensity of resistance (Trape, 2001).

The first reported cases of CQ-resistant infections were confirmed in non-immune tourists in Kenya and Tanzania in 1978, followed by semi−immune Kenyans in 1982 (Shretta et al., 2000). According to Annual Report: Diagnosis, Treatment and Prevention of Malaria: Nairobi, Kenyan Ministry of Health, 61–80% of isolated parasites were resistant to CQ, and 30% of cases treated with CQ experienced clinical treatment failure (Shretta et al., 2000). According to a study, 92% (22 of 24) of isolates assessed for CQR carried the point mutation, Asn to Tyr in *pfmdr-1* codon 86, which was the most frequently reported sequence variation related to CQR in Africa. Furthermore, 83% (20 of 24) had an Asp to Tyr mutation, and both mutations were observed in 82% (18 of 24) of the isolates. Both alleles have reported association with CQR. In addition, 75% (18 of 24) of samples each had polymorphisms on both *cg2* and *pfmdr-l* (Omar et al., 2001). Polymorphisms in the *cg2* gene, located in the 36-kb region on chromosome 7, are also related to CQR. This gene is characterized by 12 point mutations and three length polymorphisms, namely, kappa, gamma, and omega, and are associated with clones encompassing CQR phenotypes (Viana et al., 2006). Durand et al. (1999) used DNA sequencing to confirm the association of *cg2* with CQR. It was found that the *cg2* genotype including identical κ14 repeats and particular ω16 repeats displayed a strong association with CQR in all *P. falciparum* isolates from the African countries included in the study. CQR was heightened during the 1980s but continued to be the first-line treatment for uncomplicated malaria. In 1998, revised guideline for malaria treatment officially replaced CQ with a combination of SDX and PYR (Shretta et al., 2000).

In Tanzania, CQR was first demonstrated in semi-immune citizens in 1982 and in 1983 resistance spread and was reported at 34% among a Zanzibar school population. Furthermore, studies between 1982 and 1985 in various Tanzanian revealed a 20% average *in vivo*-resistant rate in schoolchildren (Trape, 2001). Consequently, CQ was the official first-line antimalarial drug for uncomplicated malaria until the end of July 2001. The parliament of Tanzania was notified by the Minister of Health, during the 2002–2003 budgetary session, that a decision was made to suspend CQ as the first-line antimalarial drug. The grounds for that decision were based on researched evidence of high cure-rate failure observed for CQ, of approximately 60% (Mubyazi and Gonzalez-Block, 2005).

By 1983, CQR had been reported in several sub-Saharan countries such as Madagascar, Burundi, Sudan, Uganda, Zambia, Comoros, Burkina Faso, Malawi, and Mozambique (Nuwaha, 2001).

The prevalence of CQR in Malawi by 1992 was an estimated 85% (Kublin et al., 2003), therefore prompting Malawi to be the first country in sub-Saharan Africa to terminate the routine use of CQ in 1993. This was supported by the increased failure of CQ treatment and its inability to produce sufficient clinical and hematological recovery. In 1990, more than 80% of Malawian children treated with CQ presented high-level parasitological resistance (Bloland et al., 1993). The removal for CQ in Malawi was supplemented by reduced prevalence of the CQR molecular marker, *pfcrt* T76, from 85% to 13% between 1992 and 2000 (Kublin et al., 2003). Widespread sensitivity to CQ was observed in Malawi after the removal of CQ as the first-line drug for malaria treatment. In 2005, a clinical trial estimated CQ efficacy at 99%, 12 years after removal. Furthermore, CQ susceptibility was maintained when used for recurring malaria episodes (Takala-Harrison and Laufer, 2015).

In 1999, a 90% prevalence of the *pfcrt* K76T mutation was identified in infected Mozambican children. A trial in southern Mozambique estimated an average of 47% for CQ’s clinical efficacy between 2001 and 2002. Furthermore, another study revealed more than 90% occurrence of the mutant CVIET haplotype in the same area (Thomsen et al., 2013). This led to the abandonment of CQ treatment for malaria in 2003 (Galatas et al., 2017).

By 1984, CQR was observed in countries including Gabon, Angola, Namibia, Senegal, Zimbabwe, and South Africa (Nuwaha, 2001). A study in Senegal examined the prevalence of *pfcrt* to determine the molecular level of CQR. A high prevalence of single *pfcrt* CVMNK wild-type haplotype was observed in the study, and the data suggested that increased prevalence of *pfcrt* wild types is occurring country-wide (Ndiaye et al., 2012). The use of CQ was abandoned by health authorities in 2003 (Trape et al., 1998). In Zimbabwe, 50% of the population reside in malaria-risk areas, and malaria transmission occurs in 51 out of 59 administrative regions (Mlambo et al., 2007). Until 1983, there were no cases of CQR in Zimbabwe; however, in 1984, the first seven cases were reported in Zambezi Valley. CQR was initially confined to the Zambezi Valley, but by 1990, it was clear that the problem had spread throughout the country (Makono and Sibanda, 1999). According to a study facilitated in the Njelele area of Gokwe following a malaria outbreak, 83% of infections were CQ resistant, with only 3% of cases responding to CQ treatment (Mharakurwa and Mugochi, 1994). The establishment of maintainable national surveillance approaches was necessary to counteract the increasing CQR and monitor its spread throughout the country (Makono and Sibanda, 1999). The clinical failure of CQ was exceptionally high and, thus, only remained the drug of choice until 2000 (Mlambo et al., 2007).

Since 1932, South Africa had faced sporadic malaria epidemics, with KwaZulu Natal, Mpumalanga, and Limpopo bearing the greatest burden within the country (Knight et al., 2009). In 1985, KwaZulu Natal had its first report of *in vitro* CQR, which spread under persistent CQ pressure, thereby leading to an escalation in cases and treatment failures. In spite of being treated with CQ four times, 3% of malaria-treated patients remained positive (Ukpe et al., 2013). CQ was replaced and no longer used as the first-line drug for malaria treatment by February 1988 (Knight et al., 2009). Malaria parasites stayed susceptible to CQ in Mpumalanga and Limpopo until the mid-to-late 1990s. The number of CQ-resistant parasites escalated, thus prompting the replacement of CQ as the drug of choice in Mpumalanga and Limpopo in 1997 and 1998, respectively (Maharaj et al., 2013).

## The Spread of Sulfadoxine–Pyrimethamine Resistance in Sub-Saharan Africa

Africa, most predominantly, sub-Saharan Africa, still remains a home to malaria, bearing high morbidity, mortality, and risk of transmission, in spite of significant reduction in malaria incidence in the region, with children and pregnant women being the most vulnerable (Zareen et al., 2016). Malaria in pregnancy is estimated to cause hundreds of thousand infant deaths every year (Kajubi et al., 2019). Therefore, in 2012, WHO recommended SP as an intermittent preventive treatment (IPT) for fetal malaria prevention in pregnant women across malarious regions (Capan et al., 2010). However, efficacy of chemoprophylaxis with SP was halted by poor compliance of patients, which has led to emergence of drug-resistant strains of *P. falciparum* (Oyibo and Agomo, 2011).

The SP is a combination comprising SDX, which inhibits DHPS, a bifunctional protein that interacts with hydroxymethylpterin pyrophosphokinase, and PYR, which inhibits DHFR bifunctional protein (Nzila et al., 2000); it, therefore, synergistically inhibits the folate biosynthesis pathway of *P. falciparum* (Ahmed et al., 2006).

**FIGURE 2:**
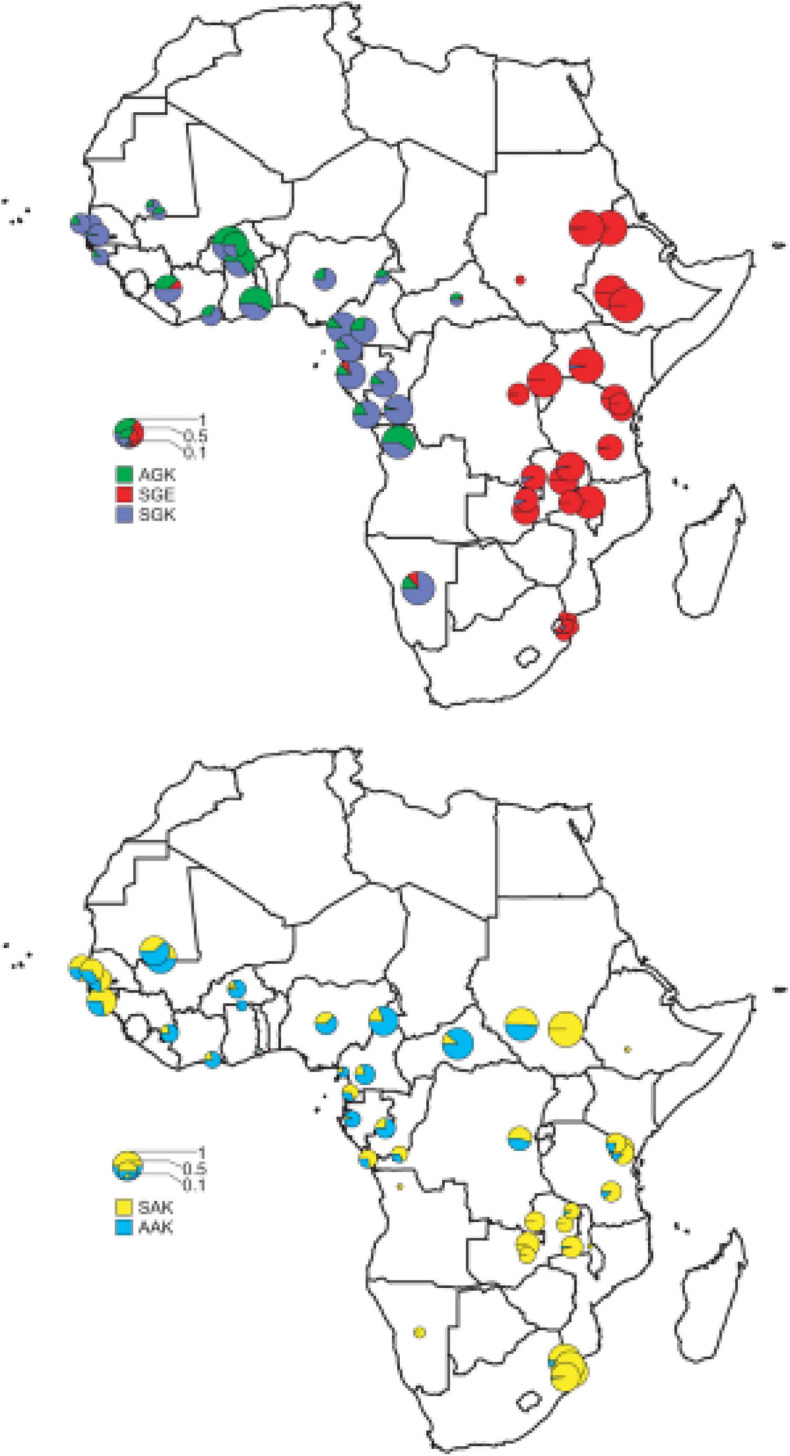
The distribution of the major *pfdhps* alleles across sub-Saharan Africa. Resistant alleles; the upper map shows the relative proportions of the three major resistance alleles, SGK, AGK, and SGE. Wild-type alleles; the lower map shows the ratio of SAK and AAK alleles among wild-type *pfdhps* alleles (Pearce et al., 2009).

The molecular basis of *Plasmodium* resistance to SP is shown to be point mutations (Gatton et al., 2004) at seven sites in the DHPS gene (*dhfr*) that confer resistance to PYR and five sites in the DHFR (*dhps*) gene that confer resistance to SDX. Different combinations of mutations in each gene lead to varied resistance levels of SP (Pearce et al., 2003). For *pfdhps*, several point mutations—Ser → Ala at codon 436, Ala → Gly at codon 437, Lys → Glu at codon 540, Ala → Gly at codon 581, and Ser → Phe at codon 436—coupled with either Ala → Thr or Ala → Ser at codon 613 confer resistance to SDX. For *pfdhfr*, a point mutation of Ser → Asn changes at position 108 (S108N); resistance mutations Asn → Ile at codon 51 and/or Cys → Arg at codon 59 (C59R) and a Ser → Thr mutation at position 108 (S108N) with an Ala → Val change at position 16, synergistically confering PYR resistance in *Plasmodium* species (Kublin et al., 2003). The DHPS domain of the bifunctional 7,8-dihydro-6-hydroxymethylpterin pyrophosphokinase (PPPK)-dhps enzyme for SDX and the DHFR domain of the bifunctional DHFR-thymidylate synthase (DHFRTS) enzyme for PYR are key targets of SP drug, as these enzymes are responsible for the folic acid biosynthetic pathway (Basco et al., 1998). After approximately 50 years of SP’s introduction, kinetics in children is still poorly understood; therefore, dosing is predominantly based on age for practical reasons, which may result in the administration of inaccurate quantities (De Kock et al., 2018). Increasing resistance to SP is linked to the stepwise acquisition of specific point mutations at specific codons in the *dhfrdhps* genes, which alter the drug-binding sites of SP (Oyibo and Agomo, 2011). However, the prophylaxis treatment of malaria was selected for double and triple mutations in *dhps* and *dhfr* genotypes, respectively, which are implicated in *Plasmodium* resistance to SP across sub-Saharan Africa (Capan et al., 2010; Sridaran et al., 2010). Accumulation of mutations within the genes (N51I, C59R, and S108N) in *pfdhfr* and mutations in codons A437G, K540E, and A581G within *pfdhps*, which confer resistance of *Plasmodium* to SP (Takala-Harrison and Laufer, 2015), has spread across sub-Saharan Africa (Figure 2).

In Benin, *pfdhps*, which confers resistance to SDX, contained a mixture of wild-type and mutant alleles (A437 and G437). High mutated alleles of *pfdhfr* were reported for codons N51I, C59R, and codon S108N. The *pfdhfr* (N51I, C59R, and S108N) proportion of single, triple, and quadruple mutations was very high in the study population (Ogouyèmi-Hounto et al., 2013). In Nigeria, parasite clearance was observed in pregnant women using SP (Kalanda et al., 2006), with mutations, N51I, C59R, S108N, and the triple mutation conferring a high level of *Plasmodium* resistance to SP (Iriemenam et al., 2012). A study conducted in Mali between August 2004 and January 2006 and in Ghana in 2003 supported the use of SP + AS (SP and amodiaquine) to treat young children with uncomplicated *P*. *falciparum* malaria and IPT in pregnant women (Tagbor et al., 2006; Tekete et al., 2011; Maiga et al., 2015). Additionally, in Mali, prevalence of each of the three *pfdhfr* mutations at codons N51I, C59R, and S108N was observed in 2007. Notably, *pfdhps* mutations at codons A437G and K540E as well as *pfdhfr* triple and *pfdhps* A437G quadruple mutations were present (Dicko et al., 2010). SP resistance was still quite low (Figueiredo et al., 2008; Dicko et al., 2012) in 2008 but rapidly increased in Mali in 2010 and Burkina Faso in 2012 (Coulibaly et al., 2014). The triple mutant *pfdhfr* in Africa evolved from the southeast Asian lineage (Coulibaly et al., 2014), while the N51I + S108N double-mutant *pfdhfr* alleles are a local origin. Both the *pfdhps* double mutants (Gly-437 and Glu-540) and the *pfdhfr* triple mutants were individually associated with SP treatment failure in children aged less than 5 years, with *pfdhps* and *pfdhfr* quintuple mutations being highly associated with SP treatment failure in Ibadan, Nigeria (Happi et al., 2005). The *pfdhfr* N51I, C59R, S108N triple mutation had a prevalence close to 100% in Cameroon, western Africa. The most frequent *pfdhps* mutation there was A437G, with a prevalence of 76.5% and had a higher significant prevalence in pregnant women with SP uptake (Chauvin et al., 2015).

Ghana, a western African country, in 2003 also recorded the quadruple mutation in *pfdhps* A437G and *pfdhfr*—N51I, C59R, and S108N (Marks et al., 2005)—but SP was still effective in 2001 for treating uncomplicated *P. falciparum* malaria whether as monotherapy or combined therapy in Cameroon (Basco et al., 2002). Meanwhile, emergence of SP combination resistance was sustained for many years before introduction of ACTs as a frontline antimalarial in Africa (Marks et al., 2005). There is a strong relationship between protective efficacy and the frequency of resistance mutations, as negative correlation exists between SP prophylaxis efficacy and parasite mutations, indicating that as resistance increases, protective efficacy decreases. This decreased efficacy of SP on IPTi was reported across Ghana and Gabon where decreased efficacy was linked to *pfdhfr* triple mutation (Griffin et al., 2010). A study in Niger showed a high proportion of *pfdhfr* N51I, C59R, and S108N haplotypes associated with resistance to PYR and *pfdhps* S436AFH and A437G mutations associated with reduced susceptibility to SDX (Grais et al., 2018). In 2004, a molecular genotyping study conducted in Laine, Guinea, found three *pfdhfr* mutations in 85.6% patients and quintuple *pfdhfr*/*pfdhps* mutations in 9.6% showing a progressive increase in resistance to SP (Bonnet et al., 2007). No *pfdhfr* I164L or *pfdhps* A581G mutations were found, but *pfdhps* K540E mutation was found in Mali, with a very low prevalence in Mali and Burkina-Faso (Coulibaly et al., 2014).

In Central Africa, between 2002 and 2003, there was adequate clearance of parasitemia in children treated with SP with low presence of *pfdhfr* S108N, *pfdhfr* triple, and *pfdhfr*/*pfdhps* quadruple mutation genotypes. This shows that studies conducted a decade ago still showed some SP efficacy despite accompanying resistance genotypes. The parasite population of Uige, Angola, revealed high frequency mutations in *pfcrt*, *pfdhps*, and *pfdhfr*, conferring resistance to CQ and SP (Coulibaly et al., 2014).

The frequency of the double A437G, K540E mutant *pfdhps* allele (conferring SDX resistance) increased from 200% to 300%, and the triple mutant *pfdhfr* (N51I, C59R, and S108N) allele (conferring PYR resistance) increased by 37–63% in Tanzanian population when SP was used as a first-line antimalarial (Malisa et al., 2010). A study conducted in 2002 in Tanzania confirmed the presence of *pfdhfr*/*pfdhps* mutations even when SP was efficient (Mbugi et al., 2006). SP resistance was reported across regions of Tanzania where the triple mutations (N51I, C59R, and S108N) were constantly predominant across all the regions, implying a high level of SP resistance (Schönfeld et al., 2007; Bertin et al., 2011; Baraka et al., 2015), which was corroborated by same result in Benin, a western African country (Bertin et al., 2011). No relationship exists between the number of mutations and the degree of parasitological resistance (Eriksen et al., 2004). Additionally, Tanzania not only reported SP failure, but its prophylaxis was of no significant effect in northern Tanzania in 2006, as triple mutations previously reported in other parts of Africa were also detected (Gesase et al., 2009). The highest mutations of *pfdhfr* and *pfdhps* genes were predominantly distributed across eastern and southern Africa where SP use has been the highest (Enosse et al., 2008; Griffin et al., 2010). In 2005, northwest Ethiopia displayed a gradual reduction of individual *pfdhps*/*pfdhfr* mutations; triple, quadruple, and quintuple mutations were observed after 5 years of SP withdrawal as a frontline antimalarial (Hailemeskel et al., 2013), but quintupled in western Kenya between 2008 and 2009 (Iriemenam et al., 2012). Moreover, by 2000, the East African Network for Monitoring Antimalarial Treatment (EANMAT) reported SP failure across East Africa, with Rwanda and Burundi exceeding the critical 25% value SP failure rates (Rapuoda et al., 2003); this could be due to a low uptake of SP by pregnant women due to weak health system and sub-optimal implementation policy, among other factors (Martin et al., 2020). The *pfdhps* A581G and *pfdhfr* I164L mutations, which confer SP resistance, have been found to be unevenly distributed across East Africa, suggesting a high level of SP resistance, as indicated by the prevalence of the K540E mutation, found across East Africa (Naidoo and Roper, 2011).

Zimbabwe reported high prevalence of *pfcrt* (K76T) to be 64%, 82%, and 92% in Chiredzi, Kariba, and Bindura, respectively. On the *pfdhfr* locus, the presence of triple mutations at codons N51I, C59R, and S108N was approximately 50% across the three locations, therefore indicative of widespread prevalence of molecular markers associated with CQ and PYR resistance in Zimbabwe (Schleicher et al., 2018). KwaZulu Natal in South Africa had reported SP resistance in 2000 and thus changed its malaria policy to the use of ACTs for malaria treatment (Knight et al., 2009).

In 2010, the quadruple mutation (A437G, N51I, C59R, and S108N) haplotypes were widespread throughout sub-Saharan Africa, particularly in Uganda, Mali, Kenya, and Malawi (Takechi et al., 2001; Osman et al., 2007), but the quintuple and sextuple mutants were found only in specific regions of Africa (Walker et al., 2017). The quintuple mutants, which plateaued in East and Southern Africa, were relatively rare in west Africa and parts of central Africa. Estimates of the prevalence of the *pfdhps*-A581G mutation suggest that the sextuple haplotype had become established in Rwanda, Burundi, D R Congo, southwestern Uganda, northwestern Tanzania, southeastern Kenyan, and northeastern Tanzanian borders.

Genotyping of *pfdhfr* responsible for PYR resistance showed no mutation in codons A16V, C59R, or I164L, while there was 84% mutation at codons N51I and S108N (Osman et al., 2007). Additionally, linkage exists between CQ mutation, *pfcrt* K76T, SDX mutation, *pfdhps* K540E, and PYR mutation *pfdhfr* (N51I and S108N), thus conferring parasite resistance against both CQ and SP. The C59R mutation observed in Mali, Kenya, and Malawi was, however, absent in Sudan in spite of 80% prevalence of *pfdhps* K540E. *Pfdhfr* N51I and S108N occurred in more than 80% (Osman et al., 2007). The A437G mutation at *pfdhps* and the triple mutation (N51I, C59R, and S108N) at *pfdhfr* associated with SP resistance is concurrent with those found even outside sub-Saharan Africa (Checchi et al., 2002). In six countries of Eastern and Southern Africa, more than 90% prevalence of *pfdhps*-K540E was implicated in SP resistance (Iriemenam et al., 2012). However, countries like Cameroon, Ghana, Zambia, Mozambique, Mali, and Zimbabwe had three doses of SP in their policy for all pregnant women, which seemed efficacious with resistant genotypes concurrently found in these countries (Mayor et al., 2008; Maiga et al., 2011; Kayentao et al., 2013). The quadruple mutation was uncommon compared with triple mutation, which confers a major resistance to SP in Africa (Peters et al., 2007). Conclusively, a systematic analysis conducted in African countries between 1996 and 2006 reported delayed clearance alluding to SP treatment failure (ter Kuile et al., 2015).

## Combating Chloroquine and Sulfadoxine–Pyrimethamine Resistance

Several publications confirm the widespread resistance of CQ and SP, with substantial occurrence in Asian and African countries. The reasons for this hinged on various factors, which include, but is not limited to, the overuse of these antimalarial medications, underdosage of the medications in the treatment of active malaria infection, and high susceptibility of the parasite to adapt at the genetic metabolic levels rapidly (Hyde, 2007). General preventive as well as some of the specific measures have been considered as ways to combat further spread of antimalarial resistance.

### Vector Control

Vector control makes use of ITNs/long-lasting insecticidal nets (LLINs) and IRS, which are the mainstay of malaria control. These two strategies have proven useful in the reduction of local malaria transmission by protecting susceptible individuals against infective mosquito bites, thereby leading to a reduction in the level of malaria intensity. When sleeping under the net, the net serves as a means of preventing mosquito contact or bite. Reducing the vector population through this means can indirectly serve as a measure to mitigate the spread of CQ- and SP-resistant *Plasmodium*. In fact, since 2007, the WHO has recommended universal coverage with ITN (Macintyre et al., 2011; World Health Organisation (WHO), 2011). IRS has also been reportedly effective in reducing the challenge of transmission. It serves as a means to terminating these vectors, thereby reducing the possible malaria morbidity and mortality (World Health Organisation (WHO), 2011). When vector control measures are well applied in combination, it is evident that the outcome of malaria transmission (CQ/SP resistant in this case) will be mitigated (World Health Organisation (WHO), 2010; Thu et al., 2017).

### The Introduction of Artemisinin-Based Combination Therapies

An important strategy continues to be the use of effective antimalarials. However, this is threatened by the extensive resistance observed in the parasite toward the most affordable classes of drugs, thus highlighting the need for novel antimalarials (Winstanley et al., 2004) and ultimately a vaccine. The widespread resistance of the known frontline antimalarial drugs—CQ and SP—in April 2002 led to the introduction of ACTs as the first-line treatment for malaria by the WHO. Notably in the treatment of *P. falciparum*, it is recommended that two or more drugs must be combined. The combined drugs must have different modes of action. Since artemisinin-based compounds (dihydroartemisinin, artemether, and artesunate) are fast acting, they are usually combined with other classes of antimalarial with long half-life such as mefloquine, amodiaquine, lumefantrine, and piperaquine (Bell and Winstanley, 2005). The ACTs, which come in various combinations, is used to replace CQ and SP in order to prevent further increasing morbidity and mortality resulting from the observed resistance in the initial use of first-line medications (World Health Organisation (WHO), 2003; Dalrymple, 2009). The high efficacy and ability to interrupt the development of resistance supported the use of ACTs as the core of malaria treatment (Laxminarayan et al., 2006). The artemisinin component of ACTs has the ability to reduce parasitemia; thus, the progression from uncomplicated malaria to a severe form of the disease can be prevented by early treatment with ACTs, consequently reducing the amount of severe malaria cases and the rate of malaria mortality (Ndeffo Mbah et al., 2015). By June 2008, most malaria endemic nations have adopted the ACTs as its front-line antimalarial medication (World Health Organisation (WHO), 2008). Part of the measures put in place to avoid similar medication resistance as seen in CQ and SP is the disallowance of artemisinin monotherapy medications. This was part of the World Health Assembly resolution of 2007 (World Health Organisation (WHO), 2008).

### Medication Adherence and Avoiding Self-Medication

Among humans, there are a variety of beliefs that can impact our behavior significantly. These beliefs are scarcely changed and can therefore affect adherence to antimalarials such as ACTs, thus highlighting the need for patients’ beliefs to be considered when care is offered (Amponsah et al., 2015). Numerous interventions have been developed and employed in malaria endemic areas to promote adherence to medications. Clear guidelines to better adherence can be achieved through deeper understanding of the relative efficacy of each intervention (Fuangchan et al., 2014). The understanding of malaria treatment could be improved by community education, such as training of dispensers and promoting public awareness, thus improving adherence to antimalarials (Denis, 1998). To ensure malaria treatment approaches continue to be effective and improve the possibility of a positive outcome, medication adherence should be evaluated regularly, thereafter used as tools to encourage appropriate action, if necessary (Gerstl et al., 2015).

The CQ/SP resistance developed partly due to widespread presumptuous use of these medications without prior objective confirmation of the diagnosis. Due to the poverty and economic setting of many malaria endemic countries especially in sub-Saharan Africa, most people self-medicate and thereby inappropriately use CQ/SP (Idowu et al., 2006). To this end, the WHO essentially recommended and introduced affordable point-of-care rapid diagnostic tests for definitive malaria testing and diagnosing before the usage of an antimalarial even in under-resourced settings. This is expected to be accompanied by correct and complete usage of the antimalarial especially in this era of ACTs (White et al., 2009; Thu et al., 2017).

### Combating the Counterfeit Medication Market

Generally, counterfeit medication presents a great challenge globally, thus contributing to the burden presented by the development of antimalarial drug resistance. Studies done in Eastern DR Congo showed that majority of the CQ has its active ingredient to be significantly underdosed while the SP was overdosed in terms of the active ingredients (Atemnkeng et al., 2007). A similar study in Nigeria corroborated the fact that the menace of counterfeit medication is huge, with 50% of CQ dispensed by street vendors underdosed of the active ingredients (Idowu et al., 2006). In light of this, the development of CQ and SP resistance is substantiated. However, going forward, this can be combatted by action at the policy level. Enacting the laws guiding counterfeit medications and strengthening of the institutions responsible will go a long way to reduce, if not totally remove, counterfeit medications in the society. This can mitigate the CQ/SP resistance in the long run (Yadav and Rawal, 2016). Another way to combat the problem of counterfeit medication is the provision of novel, effective, and affordable antimalarial drugs. This issue is addressed by the Medicines for Malaria Venture, whose aim consists of the discovery, development, and the facilitation of those antimalarials (Medicines for Malaria Venture [MMV], 2020). MMV is a non-profit Swiss foundation, officially introduced in 1999. It was one of the first public–private partnerships created to address the drug innovation gap of malaria (Bathurst and Hentschel, 2006). Developing medicines for children addresses the population most at risk of dying from malaria, thus serving as motivation for MMV to develop child-friendly formulations of current antimalarials and to develop next-generation medicines (Medicines for Malaria Venture [MMV], 2020). In 2009, Novartis and MMV developed an artemether–lumefantrine formulation, called *Coartem*^®^ *Dispersible*, adapted to cater to the needs of children affected by *P. falciparum* malaria and was the first successfully co-developed product launched by the MMV, in which 400 million products were distributed (Premji, 2009; Medicines for Malaria Venture [MMV], 2020). MMV continues to develop and improve access to affordable medicines to lessen the global burden of malaria.

### Reintroduction of Chloroquine in the Chloroquine-Sensitive Environment

Various studies have shown that with replacement of CQ/SP as the first line of treatment and subsequent replacement with ACTs, there was notable decrease in the prevalence of pfcrt K76T present in such communities. This was first demonstrated in Malawi in 2004, with the prevalence of pfcrt K76T reaching an undetectable level (Plowe, 2014).

Similar studies have validated this point in China and Zambia, with the outcome of both studies showing a resurgence of CQ sensitivity (Mwanza et al., 2016; Lu et al., 2017b). Considering the role of CQ/SP safety profile and affordability, this looks promising, and reintroduction of CQ/SP may be the way to go in the near future.

## Conclusion

In sub-Saharan Africa, where the burden of malaria infection is great, one of the core challenges for malaria control is the emergence and spread of antimalarial drug resistance. Although other strategies are employed for malaria control such as ITNs and IRS, which are the foundation of the malaria control agenda, antimalarial drugs are essential to reduce transmission. However, because of their accessibility and affordability, CQ and SP—both first-line drugs in the past—were used excessively, which resulted in the establishment of resistance, which in turn hindered malaria control in sub-Saharan Africa. This prompted the introduction of ACTs, which decreased the pressure on CQ and SP in some regions of sub-Saharan Africa, thus encouraging their reintroduction in those regions. Ongoing antimalarial drug resistance should be closely monitored in sub-Saharan Africa to prevent the establishment and spread of resistance and to detect the return of sensitivity, which could result in the possible reintroduction of specific antimalarials.

## Author Contributions

AR: writing, editing, and collation. LM, OO, and OA: writing and editing. RM: supervision and editing. MA and MO: conceptualization, supervision, editing, and funding.

## Conflict of Interest

The authors declare that the research was conducted in the absence of any commercial or financial relationships that could be construed as a potential conflict of interest.
